# Comparative physiological plasticity to desiccation in distinct populations of the malarial mosquito *Anopheles coluzzii*

**DOI:** 10.1186/s13071-016-1854-1

**Published:** 2016-11-02

**Authors:** K. Hidalgo, D. Siaussat, V. Braman, K. R. Dabiré, F. Simard, K. Mouline, D. Renault

**Affiliations:** 1 UMR CNRS 7261, Institut de recherche sur la Biologie de l’Insecte, Université François Rabelais, Faculté des Sciences et techniques, Avenue Monge, Parc Grandmont, Tours, 37200 France; 2 Université de Rennes 1, UMR CNRS 6553 Ecobio, Campus de Beaulieu, 263 Avenue du Général Leclerc, CS 74205, Rennes Cedex, 35042 France; 3 MIVEGEC, UMR IRD 224-CNRS 5290-Université de Montpellier, Institut de Recherche pour le Développement, 911 Avenue Agropolis, BP 64501, Montpellier cedex 5, 34394 France; 4 Department of Sensory Ecology, UMR 7618 Institute of Ecology and Environmental Sciences of Paris, Université Pierre et Marie Curie (UPMC), 4 Place Jussieu, Tour 44-45, 3ème étage, Paris, 75005 France; 5 Institut de Recherche en Sciences de la Santé (IRSS), Direction Régionale de l’Ouest (DRO), 399 Avenue de la Liberté, 01 BP 545, Bobo-Dioulasso, Burkina Faso

**Keywords:** Survival strategy, AKH, Dry season, Energetic reserves, Metabolomics, *Anopheles coluzzii*, Malaria, Burkina Faso

## Abstract

**Background:**

In West Africa, populations of the malaria vector mosquito, *Anopheles coluzzii*, are seasonally exposed to strong desiccating conditions during the dry season. Their dynamics strictly follows the pace of the availability of suitable larval development sites (water collections). Accordingly, mosquitoes can reproduce all year long where permanent breeding is possible, or stop reproduction and virtually disappear at the onset of the dry season when surface water dries up, like observed in temporary habitats of dry savannah areas. This highlights the strong adaptive abilities of this mosquito species, which relies at least in part, upon physiological and molecular mechanisms of specific signatures.

**Methods:**

Here, we analysed a range of physiological and molecular responses expressed by geographically different populations of *An. coluzzii* inhabiting permanent and temporary breeding sites from the north and the south-west of Burkina Faso. Four mosquito colonies, namely (i) Oursi, built from females breeding in permanent habitats of the north; (ii) Déou, from temporary northern habitats; (iii) Soumousso from south-western temporary breeding sites; and (iv) Bama, from permanent habitats of the same south-western zone, were reared in climatic chambers under contrasted environmental conditions, mimicking temperature, relative humidity and light regimen occurring in northern Burkina Faso. Female mosquitoes were analysed for the seasonal variation in their amounts of proteins, triglycerides and free-circulating metabolites. The expression level of genes coding for the adipokinetic (AKH-I) and the AKH/corazonin-related peptides (ACP) were also assessed and compared among populations and environmental conditions.

**Results:**

Our analysis did not reveal an apparent pattern of physiological and molecular variations strictly correlated with either the larval ecotype or the geographical origin of the mosquitoes. However, specific distinct responses were observed among populations, suggesting that dry season survival may rely on more complex ecological parameters at a micro-habitat scale. Interestingly, the physiological and molecular data support the hypothesis that different aestivation abilities exist among populations of *An. coluzzii* inhabiting contrasted ecological settings. In particular, the striking metabotypes differentiation and the AKH mRNA expression level observed in females from temporary northern populations may suggest the existence of a “strong” aestivation strategy in these specimens.

**Conclusion:**

Our work provides insights into the physiological and molecular basis of dry and rainy season responses in *An. coluzzii*, and highlights the important diversity of the mechanisms involved. Such results represent key data for understanding the ecophysiological mechanisms underpinning the strong adaptive potential of this malaria vector species, which undoubtedly contributes to the spreading of mosquito distribution areas in space and time.

**Electronic supplementary material:**

The online version of this article (doi:10.1186/s13071-016-1854-1) contains supplementary material, which is available to authorized users.

## Background

In several habitats, the concomitant variation of temperature and relative humidity (RH) can increase the risk of desiccation in terrestrial insects. To overcome this situation, physiological adjustments, including increased body water storage (i.e*.* metabolic water stores and bulk water), reduced body water loss rates or increased tolerance to body water loss, have been reported [1, 2]. Additional mechanisms can be triggered to limit desiccation-induced body water losses. For instance, degradation of triglycerides can participate to increase desiccation resistance, by providing a non-negligible amount of metabolic water by β-oxidation and subsequent oxidative phosphorylation [3]. In parallel, organisms can accumulate metabolites with osmoprotective functions, which can elevate hemolymph osmolality during desiccation and/or prevent damages to biomolecules and biostructures [4]. Along this line, low molecular weight compounds such as sorbitol, inositol, trehalose or proline are commonly accumulated by arthropods subjected to water stress [5, 6]. In larvae of the mosquito *Culex tarsalis*, significant increases of hemolymph trehalose and proline amounts were measured when organisms faced water stress in the form of saline exposure [7].

Adipokinetic hormones (hereafter referred to as AKH) are generally known to increase the amount of substrate metabolites for the intermediary metabolism of insects during exercise (reviewed in [8]), and play a pivotal role in the synthesis and transport of trehalose and proline from the fat body to the hemolymph [8–10]. AKH peptides control the nutrient homeostasis in general, and their involvement into the regulation of diapause metabolism has been demonstrated in the bug *Pyrrhocoris apterus* [11]. Here, we hypothesize that the multifunctional mode of action of AKH peptides could highlight the responses to desiccation in mosquitoes, by prompting synthesis of proline and trehalose, and their subsequent augmentation in the hemolymph of the organisms.

In West Africa, the drastic reduction of water precipitations and the concomitant increase of the temperature during the 6–9 months the dry season lasts highly increase the risk of desiccation in small ectotherms. Among insects, the malarial mosquito *Anopheles coluzzii* (Diptera: Culicidae; previously described as the M molecular form of *An. gambiae* (*s.s.*), see [12, 13]) is widespread throughout West Africa. Population dynamics of this mosquito fluctuates from one season to the next, therefore impacting malaria transmission. In particular, the mosquito population dynamics follow the pace of its larval breeding sites (i.e. water collections), which can be characterised as permanent or temporary [14, 15]. Hence, in areas where large anthropogenic (e.g. dams and rice fields) or natural (e.g. ponds, rivers edges, ruts, etc.) surface water collections are annually found, this mosquito can breed all year long (i.e. populations are considered as permanent). Conversely, in areas where surface water is available only during the rainy season, mosquito populations locally disappear during the dry season (i.e. populations are considered as temporary). This ability of *An. coluzzii* to endure a large range of temperature and relative humidity conditions across their larval ecotypes likely results from their capacity to express several distinct phenotypes [16], explaining, at least partially, the robustness of malaria transmission throughout West Africa from one season to the next. Consistently, at the onset of the dry season, it was established that female *An. coluzzii* may enter a different level of recurring states of summer dormancy (aestivation), characterised by suppressed reproduction and growth [17–19]. In a recent work, specimens from temporary mosquito populations were described as “strong aestivators” programmed to enter a high level of summer dormancy at the onset of the dry season [20]. Conversely, females from permanent populations were characterised as “weak aestivators”, being able to enter a dormant state only if breeding sites are not easy to reach taking them away from lay eggs and complete their gonotrophic cycle [20].

In the present study, we assessed the physiological plasticity of *An. coluzzii* females experimentally exposed to situations mimicking the climatic conditions that mosquitoes experience in their natural habitats during the rainy season (optimal reproduction and growing period) and the onset of the dry season (desiccating conditions, assumed as a stressful period for the mosquitoes). We compared the biochemical responses of *An. coluzzii* females originating from temporary and permanent larval ecotypes from two regions of the Burkina Faso (West Africa), i.e. sampled from the northern and south-western regions of this country, which can be characterised as arid and more humid, respectively. We expected that responses of females to the desiccating conditions of the dry season would differ among mosquito populations, thus depicting physiological plasticity and local adaptation to the environmental conditions of their respective microhabitat. Specifically, mosquitoes originating from the north of Burkina Faso, where dry season conditions are hasher than in the southwest, and mosquitoes from temporary breeding sites should be characterised by better abilities to cope with desiccation. We also aimed at examining what is the major factor triggering the highest seasonal plasticity to desiccation in this anopheline species: is it the geographical origin (north *vs* southwest), the larval ecotype (permanent *vs* temporary breeding-sites) or the interaction of both factors at the micro-habitat level (specific desiccation response for each population)? We hypothesised that exposure to the conditions of the onset of the dry season would trigger changes in (i) whole body energetic reserves (triglycerides and proteins amounts), (ii) the amount of circulating metabolites, and more particularly those known for their osmoprotective roles, and (iii) the expression levels of AKH peptides. Finally, we expected to find that mosquitoes programmed to engage into “strong aestivation” (i.e. those from temporary habitats) would exhibit higher physiological changes when exposed to dry conditions, and these changes would be exacerbated in mosquito population from the north of Burkina Faso because of more arid local conditions. In particular, these female mosquitoes should have overexpressed *AKH* mRNA levels, and should exhibit higher amounts of osmoprotectant compounds. Furthermore, we expected that this work highlights elements of the seasonal ecophysiology of different *An. coluzzii* populations in West Africa in order to propose innovative methods for the control of their populations at more local levels.

## Methods

### Mosquitoes

**Fig. 1:**
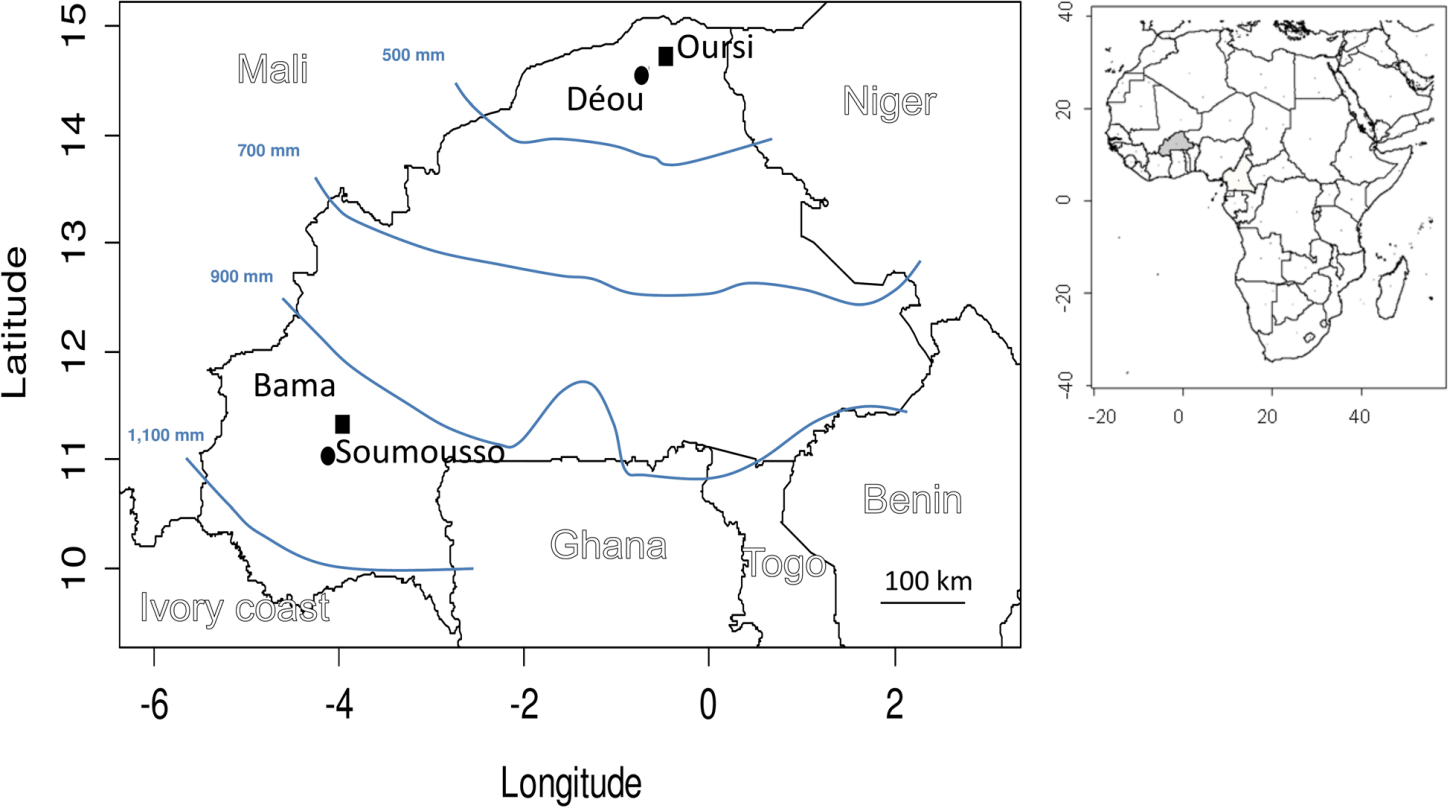
Geographical localisation of the four localities where wild *An. coluzzii* mosquitoes were collected in northern and south-western regions of Burkina Faso (West Africa). *Squares* represent permanent mosquito populations and *circles* represent temporary populations (see text). The *blue* lines are the annual isohyete (mean yearly rainfall, in mm adapted from Lind & Fensholt [62]). Insert: localisation of Burkina Faso (*grey*) in Africa

Experiments were conducted using four mosquito colonies recently derived from the progeny of wild-caught *An. coluzzii* females, identified according to PCR methods [21]. Mosquitoes were sampled from two localities in the northern, most arid part of Burkina Faso, and from two localities in the south-western part of the country (Fig. 1). For both regions, mosquitoes were sampled from one locality where permanent mosquito breeding is possible [i.e. presence of permanent mosquito breeding sites in Oursi (14°40′N, 0°27′W) and Bama (11°23′N, 4°24′W); Fig. 1), and from one locality where mosquitoes breed only during the rainy season [i.e. Déou (14°35′N, 0°43′W) and Soumousso (11°04′N, 4°03′W)]. Females were collected while at rest in human dwellings in each of the four localities, and brought back to the IRSS (Institut de Recherche en Science de la Santé) of Bobo-Dioulasso for the subsequent establishment of the colonies. At least 100 gravid females of *An. coluzzii* per locality were used to establish the colonies. The colonies were maintained under controlled conditions (27 ± 1 °C, 80 ± 10 % relative humidity with LD cycles of 12 h:12 h) for up to six generations, prior to being used for the experiments.

### Experimental conditions and mosquito rearing

**Fig. 2:**
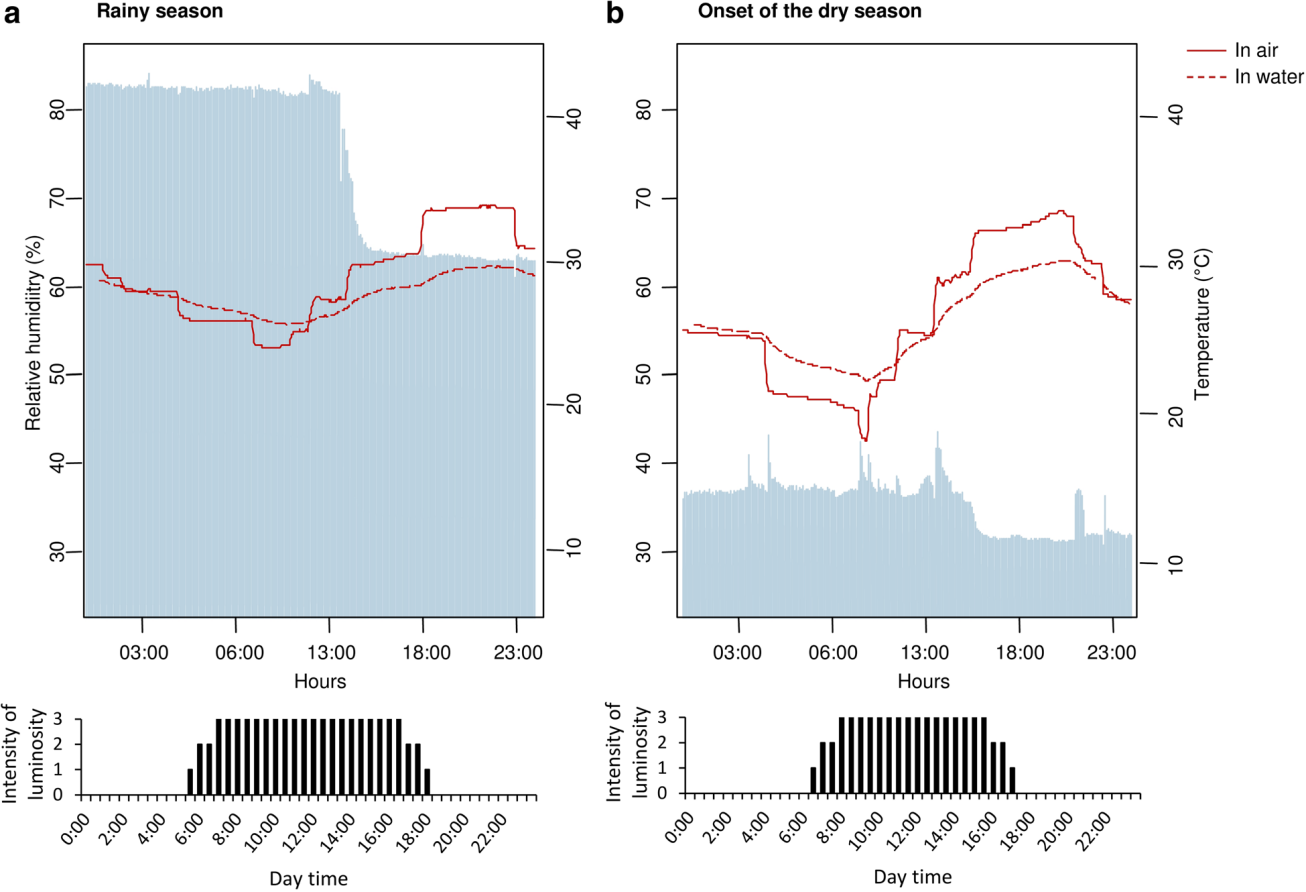
Daily fluctuation of RH (%; *blue bars*) and air temperature (°C) in the climatic chambers (*red solid lines*) and of the water temperature in the rearing trays (*red dashed lines*) in Oursi. **a** Rainy season. **b** Onset of the dry season. For each condition, photoperiod duration is represented by the *black bars* below

For each anopheline colony, females used for the experiments were obtained by breeding eggs to adults in two programmable climatic chambers (Sanyo MLR 315H, Sanyo Electric Co., Osaka, Japan). One climatic chamber reproduced the rainy season conditions, and the second one reproduced the onset of the dry season conditions (Fig. 2). Climatic chambers were switched between each rearing session to avoid “chamber effect”. From January to March 2013, three rearing sessions were run to get a sufficient number of females for the experiments. The first two rearing sessions corresponded to mosquitoes used for ecophysiological experiments (i.e. body water and dry mass measurements, total body proteins and triglycerides, and metabolomics), and the third session to mosquitoes used for qRT-PCR analyses.

The northernmost locality of Burkina Faso is characterised by stronger temperature and RH differences between the rainy and dry seasons, and, inside the dry season, by higher temperature ranges between morning (AM) and afternoon (PM), in particular at the onset of the dry season (ODS, Fig. 2). To trigger survival strategies in all the experimental populations, the climatic conditions, i.e. temperature, relative humidity (RH), and photoperiod, programmed within the climatic chambers were those from Oursi (the northernmost locality) for both the rainy season (RS) and the onset of the dry season (ODS) conditions. These climatic data, obtained from [22] and http://www.gaisma.com/, were hourly averaged over the past 30 years for a period ranging from August 1st to 31st (RS) and from December 1st to 31st (ODS). Climatic conditions were then programmed inside the climatic chambers to reproduce as closely as possible the natural daily climatic fluctuations of RS and ODS conditions. MicroLog Pro monitors (EC750, Davis Instruments, Hayward, CA, USA) were used to monitor temperature and RH inside the incubators, and inside one of the larvae rearing pans to monitor water temperature (Fig. 2). Climatic conditions of RS and ODS in the programmable chambers were consistent across the three rearing sessions (data not shown).

For each rearing session and each colony, eggs were obtained from more than 50 females. Eggs from these females were merged to achieve large sample sizes and synchronous hatching. Upon collection, eggs were directly transferred into independent plastic trays (30 × 20.5 × 6.5 cm), each containing 1 l of deionised water. Trays containing eggs were immediately transferred to the RS or ODS experimental conditions. After hatching, first instar larvae were readily transferred into new plastic trays filled with 1 l of deionised water at an optimal growing density of about 200 larvae per tray. For each mosquito population, six to nine plastic trays (i.e. about 1,200–1,800 larvae) were used for each experimental condition (RS, ODS). Every day, the trays were randomly reshuffled to avoid any positional effect within the climatic chambers. Larvae were fed daily with sprinkled ground fish food (Tetramin®) provided *ad libitum* until pupation. At pupation, pupae were collected and transferred immediately into new plastic cups (diameter 7 cm × height 8.5 cm) filled with 10 ml of deionised water, and maintained under RS or ODS conditions until adult emergence. Soon after emergence (< 1 h), males were discarded from the batches, and only teneral females were conserved. They were transferred into new plastic cups and returned to the climatic chambers. Females had permanent access to water and to a 10 % glucose solution. The glucose solution was removed 24 h before females were sampled for subsequent analyses.

For biochemical and molecular analyses, 7-day-old females (T7) were sampled, and pools of four to five (ensuring a minimum sample dry mass of 1 mg for proteins, triglycerides and metabolomics analyses) or ten (for molecular assays) specimens were immediately killed using liquid nitrogen and stored at -20 °C (for biochemical assays) or -80 °C (for molecular assays).

### Body water content and dry mass

For each population and each experimental condition (RS, ODS), fresh masses of newly emerged (< 1-h-old females, hereafter referred to as T0, *n* = 15–20 females, depending on the population), two-day-old (hereafter referred to as T2, *n* = 15–20 females, depending on the population), and 7-day-old (hereafter referred to as T7, *n* = 20–30 females, depending on the population) females were measured using a microbalance (Sartorius SE2, d = 1 μg). Specimens were then dried for three days at 60 °C before being reweighed (dry mass). Body water content was calculated for each specimen as follows: (fresh mass - dry mass)/dry mass.

### Whole body protein and triglyceride contents

Because heating at 60 °C may alter the biochemistry of the specimens, dry mass of the samples was assessed (± 1 μg, MX5 microbalance, Mettler Toledo GmbH©, Greinfense, Switzerland) after the mosquitoes were freeze-dried for 48 h (Lyovac™ GT3, Leybold AG, Hanau, Germany). Total protein content (average of *n* = 5–6 replicates per experimental condition) was assessed using a Sigma protein assay kit (Sigma Chemical Co., BCA-1), following the procedure described by [23] and revisited by [24]. Briefly, proteins were extracted in 180 μl of phosphate buffer (100 mM KH_2_PO_4_, 1 mM DTT and 1 mM EDTA, pH 7), and homogenised with two tungsten beads using a bead-beating device (Retsch^TM^ MM301, Retsch GbmH, Haan, Germany) set to 30 Hz for 1.5 min. Then, 5 μl of the supernatant was transferred into the wells of a microplate, in which 245 μl of Bradford reagent was added (B6916; Sigma-Aldrich, Saint-Quentin Fallavier, France). Samples were incubated at room temperature for 15 min and the concentration of total proteins was read at 595 nm (Molecular Devices VERSAmax Tunable). Whole body protein contents in the mosquito pools were expressed as nmoles.mg^-1^ of dry mass.

For the triglyceride assay, mosquitoes (average of *n* = 5–6 replicates per experimental condition) were homogenised in 600 μl of methanol-chloroform (1:2, v:v) solution using two tungsten beads at 30 Hz for 1.5 min (Retsch^TM^ MM301, Retsch GbmH, Haan, Germany). Then, a volume of 200 μl of ultrapure water was added (final methanol-chloroform-water solution 1:2:1, v:v:v). The samples were further homogenised and centrifuged at 4,000× *g* for 10 min at 4 °C. A 300 μl aliquot of the lower chloroform-lipidic phase was transferred into a clean 1.5 ml microtube, and dried out at 25 °C. The dry residues were then re-suspended in 100 μl of BSA-Triton X100-water solution (0.3:0.02:1, v:v:v), as described in [25]. Samples were vortexed and incubated for 10 min at 60 °C. Triglyceride content was measured using a colorimetric assay kit (Triglyceride assay kit, Cayman Chemical Company, Ann Arbor, MI, USA), following the manufacturer’s instructions. Absorbance was read at 540 nm (Molecular Devices VERSAmax Tunable) and triglyceride content was expressed in nmoles.mg^-1^ of mosquito dry mass.

### Gene expression

#### RNA extraction and cDNA synthesis

RNA was extracted from the samples (*n* = 4 biological replicates per mosquito population and per experimental condition) using TRIzol reagent (Invitrogen, Carlsbad, CA, USA) coupled to the RNeasy Kit (Qiagen, Courtaboeuf, France), before being treated with DNase I (Ambion, California, USA) in accordance with the manufacturer’s instructions. RNA amount was quantified by spectrophotometry at 260 nm (Nanodrop2000, Thermo Scientific, Darmstadt, Germany). Then, single-stranded cDNAs were synthesised from total RNAs with Superscript II reverse transcriptase (Gibco BRL, Invitrogen, Darmstadt, Germany), as described in [26], and according to the manufacturer’s instructions.

#### Real-time quantitative PCR

All real-time quantitative reverse transcription PCRs (qRT-PCRs) were conducted as described in [26]. A total of ten genes (i.e. *Actin*, *Rps13*, *Rps7*, *Rpl5*, *h3a, Cytp450, Tubulin, hsp83*, *EGFR*, and *18s*) were tested as putative housekeeping genes. Following a BestKeeper analysis [27], *Rps13* was selected as the reference gene, since its expression was stable in all samples, whatever the experimental condition, or the anopheline population tested. Further, mRNA expression of the two AKH genes *AKH-I* and *AKH-II* was monitored. AKH-II was identified as AKH/corazonin-related peptide (see [28]), and will hereafter be referred to as ACP. Specific primers (forward and reverse) for both housekeeping genes and target genes were designed using the Eprimer3 software (http://emboss.bioinformatics.nl/cgi-bin/emboss/eprimer3; Additional file 1: Table S1) referring to the NCBI genome “*Anopheles gambiae* str. PEST”.

Each qRT-PCR reaction was triplicated and consisted of 6 μl of absolute Blue SYBR Green Fluor (Roche Molecular Systems Inc., California, USA), 2 μl of cDNA (25 ng.μl^-1^), 0.5 μl of each reverse and forward primers (10 μM), and 3 μl of RNA-free water. The cycle threshold values (Ct-values) for both reference and target genes were determined using the Light-Cycler® 480 software (Roche, Meylan, France). The average Ct value of each triplicate was used to normalise candidate gene expression levels to the geometric mean of the reference gene level using the Q-Gene software [29].

### Metabolic profiling

Extraction was conducted on freeze-dried samples (average of *n =* 8–9 biological replicates per mosquito population and per experimental condition). We used the analytical procedure described in [30] for the preparation of the samples. A 10 μl volume of arabinose (3 mM, internal standard) was added to each sample to monitor the reliability of the metabolite quantification. A gas chromatograph-mass spectrometer (GC-MS) platform (Thermo Fischer Scientific Inc, Waltham, MA, USA) was used for metabolite quantification (report to [30, 31] for the description of the GC-MS settings and chromatogram annotation). All samples were run under the SIM mode (electron energy: -70 eV). The GC was equipped with a 30 m fused silica column (TR-5MS, 95% 200 dimethyl siloxane, 5% Phenyl Polysilphenylene-siloxane, I.D.: 0.25 mm). One microliter of each sample was injected using the split mode (25:1). Randomised sample sequences were established for sample injection, and chromatograms were deconvoluted using *XCalibur* v2.0.7. Standard samples, consisting of 61 pure reference compounds at 1, 2, 5, 10, 20, 50, 100, 200, 500, 750, 1000, 1500 and 2000 μM were run and metabolite levels were quantified using the quadratic calibration curves for each reference compound.

### Data analysis

All statistical procedures were conducted with the *R* 3.1.1 software. Before analysis, the normal distribution (Shapiro-Wilk test) and homoscedasticity (Bartlett and Hartley tests) of the data were checked.

Variation in body water content was assessed by an ANCOVA using the two experimental conditions (RS, ODS), the four anopheline populations, and the females’ age (T0, T2, and T7) as explanatory variables. Females’ dry mass was used as an explanatory co-variable. Main variable effects and all relevant first and second order interactions were tested in full models. Further, model simplification used stepwise removal of terms, where the significance of each term was estimated using the difference in Akaike’s information criterion (AIC). Statistical significance was set to α = 0.05 and Tukey HSD procedures were used for *post-hoc* comparisons among the levels of significant factors.

Variation in females’ dry mass was examined using an ANOVA performed with the two experimental conditions (RS, ODS) and the four anopheline populations as explanatory variables. Similar procedures were used to examine variation in protein and triglyceride contents, expression levels of both AKH and ACP mRNA transcripts.

A multivariate discriminant analysis was conducted to explore differences in metabolite content of the mosquitoes between the two experimental conditions (RS, ODS) and among the four anopheline populations. Metabolite contents were first log-transformed [x = log_10_ (X + 1)] to fulfil the assumption of normally distributed residuals. Using the log-transformed data, we performed a MANOVA to examine if there were physiological differences among the different experimental groups. Class separation was further investigated using a linear discriminant analysis (LDA). Together with the multivariate analysis, two-way ANOVAs were performed for each detected metabolite, with experimental conditions and anopheline populations as explanatory variables. ANOVAs were followed, when required, by Tukey HSD *post-hoc* procedures among levels of significant factors. The statistical *P*-values were adjusted using the two-stage Benjamini-Hochberg algorithm [32] to control for false discovery rate (α = 0.05) induced by multiple comparisons. Metabolites for which no significant difference could be demonstrated between at least two experimental groups (i.e. RS *versus* ODS; 4 populations) were discarded from the discriminant analysis. Likewise, samples encompassing metabolites that were not reliably quantified (signal/noise < 10, or concentration < quantification limit) were also discarded from the analysis. The between- and within-group degrees of freedom together with the *F*-value are reported for each LDA axis. Distribution probability of the LDA was also monitored using a Monte-Carlo permutation (10,000 random permutations; *P*-value < 0.001).

## Results

### Body water content and dry mass

**Fig. 3:**
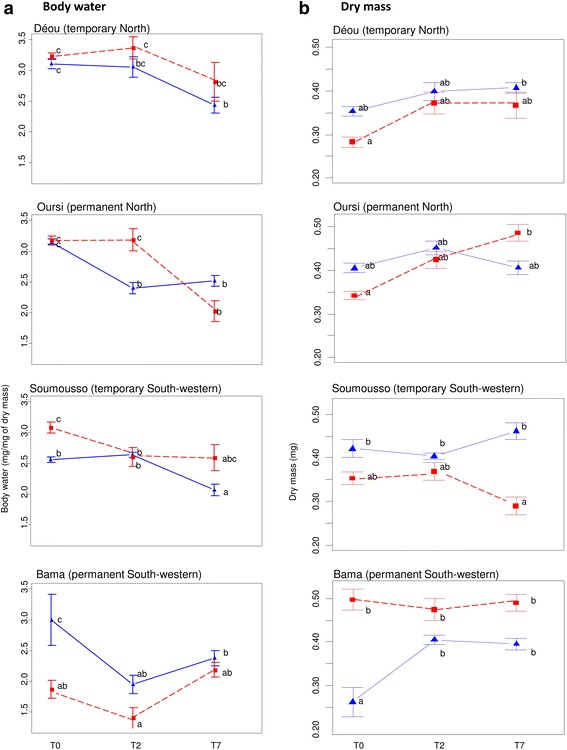
Variation in body water content (**a**) and dry mass (**b**) among females *An. coluzzii* from Déou, Oursi, Soumousso and Bama reared under RS (*solid blue lines* and *triangles*) and ODS (*dashed red lines* and *squares*) experimental conditions at 0, 2 and 7 days post-emergence. Values are means ± standard errors (SE). Different letters denote significant differences between experimental modalities (*P* < 0.05)

Body water content of mosquitoes exposed to RS and ODS conditions significantly differed among the four populations and over the duration of exposure, as supported by the significance of the three interaction terms (ANCOVA: F_(1,4)_ = 3.65, *P* = 0.006). Body water content decreased from T0 to T7 in all mosquitoes when they were maintained under RS conditions. For the ODS conditions, only the females from Oursi showed a significant decrease of their body water content over the course of the experiment (ANOVA, F_1_, *P* < 0.001) (Fig. 3a). Conversely, dry mass increased from T0 to T7 in ODS-reared mosquitoes from Oursi and RS-reared mosquitoes from Bama, but remained constant in the two other populations (Fig. 3b).

### Protein and triglyceride contents

**Fig. 4:**
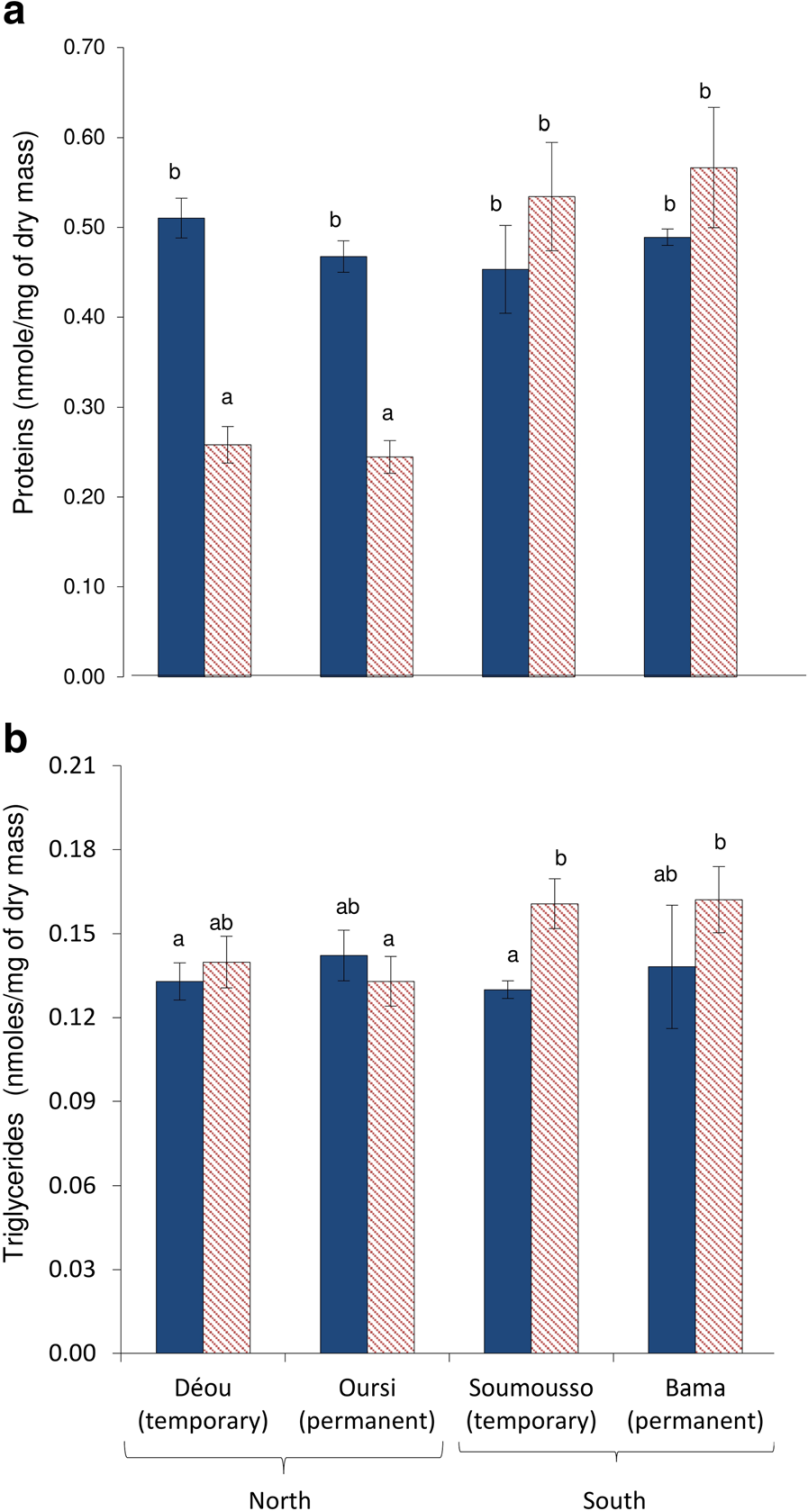
Stored proteins (**a**) and triglycerides (**b**) contents (nmoles/mg of dry mass) (mean ± standard error, SE) in 7-day-old females *An. coluzzii* from Déou, Oursi, Soumousso and Bama reared under RS (*solid blue bars*) or ODS (*dashed red bars*) experimental conditions. Different letters denote significant differences between experimental modalities (*P* < 0.05)

Protein contents were similar among the four populations reared under RS conditions (ANOVA: F_(4,1)_ = 0.35, *P* = 0.80) (Fig. 4a). In females from Déou and Oursi, protein contents were significantly reduced in ODS conditions (ANOVA: F_1_ = 10.07, *P* = 0.0004). Only mosquitoes from Soumousso showed a significant increase in triglyceride amounts from RS to ODS (ANOVA: F_1_ = 12.25, *P* = 0.025) (Fig. 4b). Otherwise, no variation in triglyceride contents was observed among populations (ANOVA: F_4_ = 0.01, *P* = 0.96) or between RS- and ODS-reared females (ANOVA: F_1_ = 1.27, *P* = 0.27; see Additional file 3: Supplementary dataset 1 for detail on raw data).

### Gene expression: adipokinetic hormone (AKH) peptide expression

**Fig. 5:**
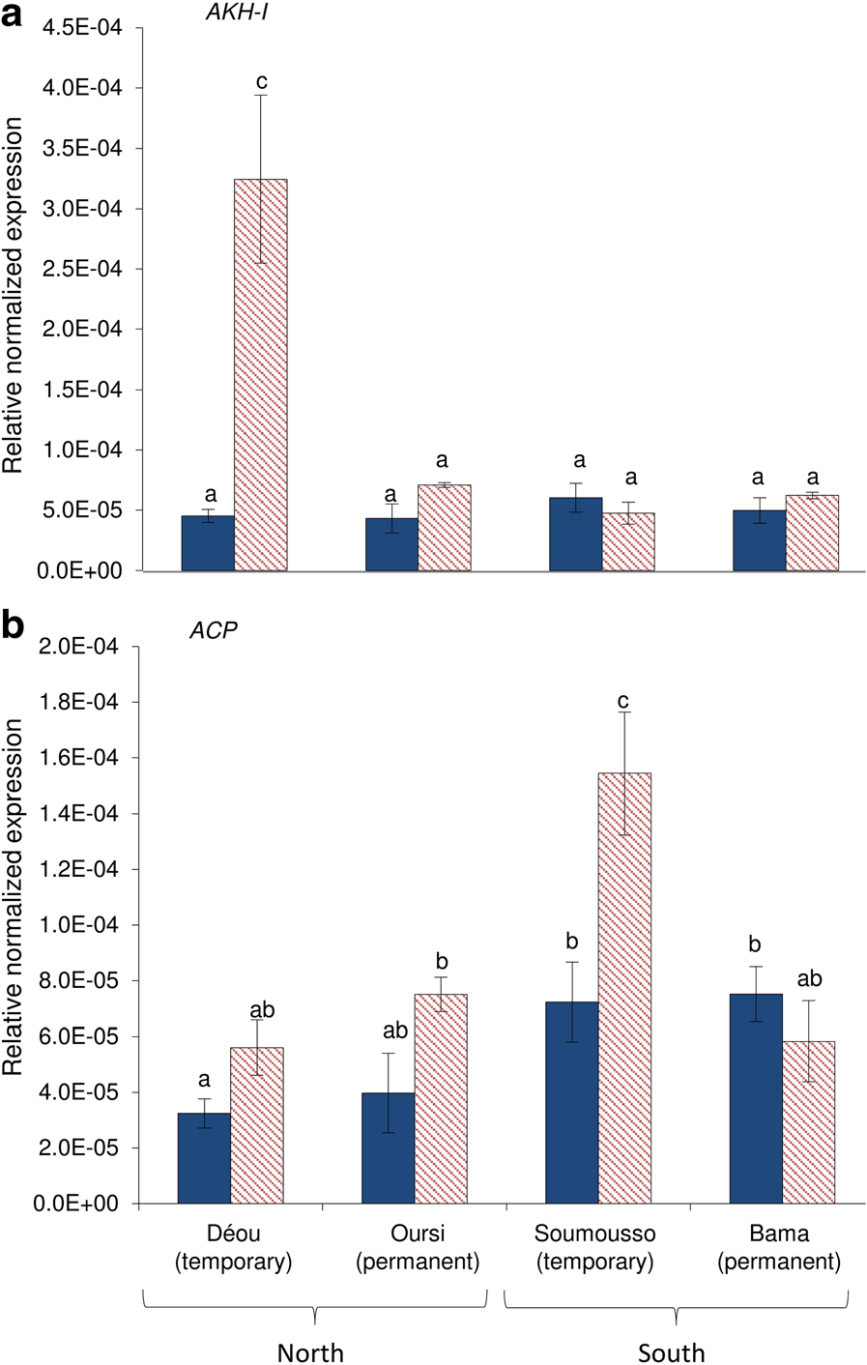
Relative normalized mRNA level expression (mean ± standard error, SE) of *AKH-I* (**a**), and *ACP* (**b**) in 7-day-old *An. coluzzii* females from Déou, Oursi, Soumousso and Bama reared under RS (*solid blue bars*) or ODS (*dashed red bars*) conditions. Different letters denote significant differences (*P* < 0.05)

Variation in the expression level of *AKH-I* and *ACP* (*AKH-II*) genes mainly depended upon the sampling locality (ANOVA: AKH-I, F_4_ = 10.62, *P* < 0.001; AKH-II, F_4_ = 3.68, *P* = 0.018). No variation was observed in the expression level of these two genes in females from Bama and Oursi. However, the amount of *AKH-I* mRNA strongly increased in ODS-reared specimens from Déou (i.e. 7.17-fold change; Fig. 5a), and in *ACP* mRNA for ODS-reared specimens from Soumousso (i.e. 2.14-fold increase compared to RS-reared females from the same locality; Fig. 5b).

### Metabolic profiling

Among the 61 metabolites from our database, 36 were accurately detected from the samples (Additional file 2: Table S2). Some of these 36 metabolites were not reliably quantified (signal/noise < 10, or metabolite concentration < quantification limit of our equipment). Therefore, three biological replicates were discarded from the analysis to successfully achieve the discriminant analysis (RS condition: one sample from Bama, one sample from Oursi; ODS conditions: one sample from Déou).

Distinct metabolic profiles were observed among the four *An. coluzzii* populations (MANOVA: F_(3,87)_ = 3.15, *P* < 0.001), and the two experimental conditions (MANOVA: F_(1,29)_ = 13.98, *P* < 0.001). In addition, contrasted metabolic profiles were found between the two experimental conditions among the different mosquito populations, as supported by the significance of the interaction terms (MANOVA: F_(3,87)_ = 2.44, *P* < 0.001).

Twenty-nine out of the 36 detected metabolites showed significant differences between at least two experimental groups, and the seven non-influential metabolites (i.e. glutamic acid, glyceric acid, leucine, lysine, methionine, phenylalanine and serine) were thus discarded from the LDA.

**Fig. 6:**
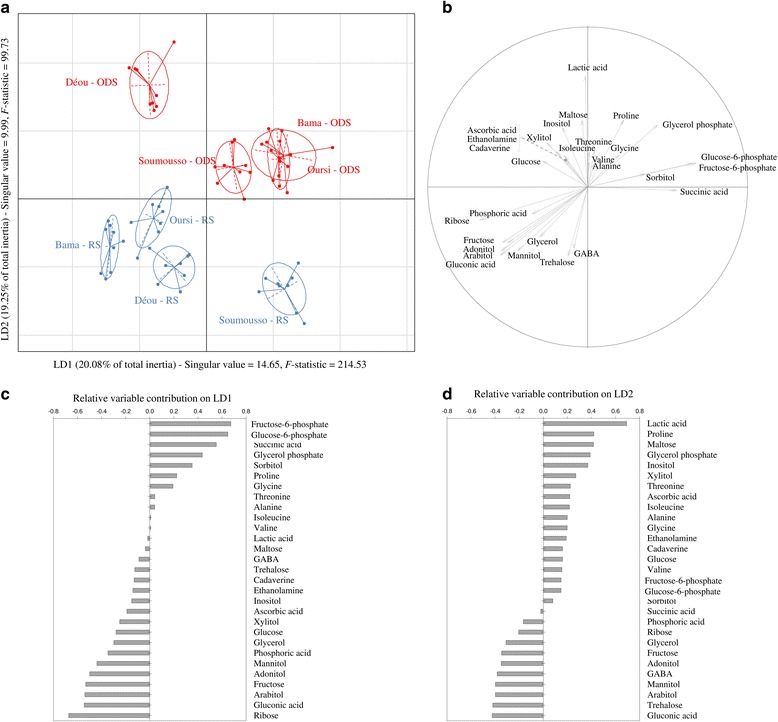
**a** Sample projection of the four populations of *An. coluzzii* females onto the first discriminant plane of the LDA. *Blue* and *red* samples represent specimens reared under RS and ODS conditions, respectively. The singular values correspond to the ratio of between-class/within-class inertias. **b** The correlations circle depicts the normed relation between each metabolite and first discriminant plane. Amino acids are identified using the European abbreviation index. **c**-**d** The relative contributions of the 29 retained metabolites to LD1 and LD2

The first axis (LD1) accounted for 20.08 % of the total inertia, and the variation among groups was 14.65 times higher than the variation within groups (Fig. 6a). LD1 mainly separated ODS-reared females from Bama and Oursi from their RS-reared counterparts. Accordingly, when exposed to RS conditions, females exhibited higher amounts of adonitol, arabitol, fructose, gluconic acid and ribose; when exposed to ODS conditions, females exhibited higher amounts of fructose-6-phosphate, glucose-6-phosphate, glycerol-3-phosphate, sorbitol and succinic acid (Fig. 6b-c).

The second axis (LD2) of the discriminant analysis accounted for 19.25 % of the total inertia, and the variation among groups was 9.99 times higher than the variation within groups. LD2 mainly corresponded to a clear cut off between RS and ODS conditions, particularly in females from Soumousso and Déou (Fig. 6a). Accordingly, females reared in RS conditions exhibited higher amounts of arabitol, GABA, gluconic acid, mannitol and trehalose (Fig. 6b, d). On the other hand, females reared in ODS conditions, and particularly those from Déou, exhibited higher amounts of glycerol-3-phosphate, inositol, lactic acid, maltose, proline and xylitol.

## Discussion

The four malarial mosquito populations used in this work were likely exposed to different environmental selective pressures in their respective larval ecotypes, eventually fostering local adaptation contributing to an explanation for the persistence of these vectors in West Africa even during the dry season. We have no genetic data for the four anopheline populations used in this work, but earlier investigations revealed that karyotype profiles of *An. coluzzii* mosquitoes in Burkina Faso differ between northern and southern populations, and between permanent and temporary breeding sites [33]. Consistent with this previous work, distinct phenotypes were observed in the four anopheline populations we used, e.g. different wing shape and size variations were measured among specimens from these populations when they were reared under ODS and RS conditions [34]. There is however no direct evidence of a possible link between genotypes and wing shape and size variations, but there are evidence that variations in mosquito wing shape underline ongoing local adaptation events, and express, at least partially, the existing genetic variation among populations [35]. The present work compared the physiological responses of females of four populations of *An. coluzzii* of distinct origin when experimentally exposed to the environmental conditions characterising the onset of the dry season in Burkina Faso. In particular, we investigated how seasonal climatic variations and geographic origin (larval ecotypes and collection area) influence (i) triglyceride and protein contents, (ii) expression level of *AKH* genes, and (iii) circulating metabolites*.*

Previous investigations performed on female *An. coluzzii* demonstrated high resistance to environmental conditions mimicking the onset of the dry season (e.g. increased temperature, decreased relative humidity and water availability [36, 37]). Increased body water amount was demonstrated to be the primary mechanism enhancing desiccation resistance in flies [38, 39], and this response was also observed in newly emerged *An. coluzzii* females [16]. Meanwhile, in the present study, body water content and dynamics of body water loss remained similar between RS- and ODS-reared females from their emergence, and until they were aged of 7 days. We speculate that supplying the females *ad libitum* with glucose and water during the experiment may have partly masked the physiological mechanisms associated with potential enhancement of desiccation resistance at ODS.

In ODS-reared specimens from Déou and Oursi (populations from the northernmost part of Burkina Faso), body protein amount was significantly reduced (about 2-fold). It is hard to decipher if this decrease in protein amount, which also likely contributed to the observed decrease in body dry mass, corresponds to a general loss in proteins, or if it could correspond to the degradation of specific proteins only (hexamerin for instance). A previous study showed that hexamerin A and 2β were characterised by drastic reductions at ODS in *An. coluzzii* females [16]. Then, amino acids resulting from the breakdown of proteins could fuel the energetic metabolism [40], and/or could be used as substrates for the synthesis of metabolites with osmoprotective functions [7, 41].

Interestingly, a distinct pattern was observed in female mosquitoes from the southwest area (Soumousso, and at a reduced level in Bama), which were mainly characterised by a slight accumulation of triglycerides when reared under ODS conditions. An elevated amount of triglycerides can enhance desiccation resistance in invertebrates, as it provides a non-negligible source of metabolic water upon oxidation [3]. A similar mechanism may have occurred in ODS-reared mosquitoes from south-western parts of Burkina Faso. Finally, the environmental conditions characterising the dry season from Oursi were used for the experiments, and these conditions are harsher than those encountered in the south of Burkina Faso (i.e. daily temperature fluctuations are higher and RH is lower than in the south). Northern populations of Déou and Oursi should be more accustomed to deal with such drastic environmental conditions, and this experimental design may have contributed, at least partially, to the different biochemical patterns measured between northern (Déou and Oursi) and south-western populations (Bama and Soumousso).

Sugar catabolism represents a prominent source of energy for fuelling the energetic metabolism during desiccation in arthropods [38, 42, 43]. In this work, levels of glycerol phosphate, glucose-6-phosphate (G6P) and fructose-6-phosphate (F6P) were more important in ODS-reared *An. coluzzii* populations sampled from localities where breeding is annually possible (i.e. Bama and Oursi). Increased amounts of G6P and F6P can be consistent with the protein breakdown we observed above in specimens from Oursi, but it can also be linked to an increase of glycogen breakdown. Both G6P and F6P are important metabolites for the glycolytic pathway, but these compounds are also initial and end products, respectively, of the non-oxidative phase of the pentose phosphate pathway (PPP), which is often elicited in insects under desiccation [44, 45]. Indeed, PPP is a major source for reductant (i.e. NADPH) and osmoprotectant production which are both used to counteract the damages caused by desiccation in insects [46–48]. We hypothesise that increased levels of both G6P and F6P at ODS depict an increase in activity of the PPP at ODS in female mosquitoes. This speculation needs to be further supported by, for instance, measurements of end products of the oxidative phase of PPP (NADPH and ribulose 5-phosphate).

Our metabolomic analyses suggest that mosquito metabotypes are rather similar among populations when they are reared under environmental parameters reproducing RS climatic conditions. In these conditions, mosquitoes were characterized by higher amounts of fructose, GABA, gluconic acid, ribose, trehalose, and by numerous polyols (adonitol, arabitol, mannitol), as compared with their counterparts exposed to ODS conditions. Trehalose together with glucose whose amounts did not vary significantly between RS and ODS conditions, are the most abundant sugars in anopheline mosquitoes [49]. High amounts of these sugars may characterize the metabolic activity of non-stressed females reared under RS conditions. Metabolic profiles of females from Soumousso differed from those of the three other populations at RS, with specimens from Soumousso exhibiting higher concentrations of F6P, G6P, glycerol-3-phosphate, sorbitol and succinic acid. Environmental conditions of the northern region of Burkina Faso (Oursi) were used in this work, where temperatures, even during the rainy season, could have been sub-optimal for *Anopheles* specimens sampled from the south-western temporary breeding site of Soumousso. Variations of the concentrations of G6P and F6P, whose roles have been discussed above, and sorbitol, are consistent with this suggestion. Indeed, sorbitol is a well-known low molecular weight compounds accumulated by insects subjected to heat or desiccation stresses [50–52]. Interestingly, metabotypes of females subjected to ODS conditions remain very similar, except for specimens originating from Déou. A striking differentiation was observed between females from Déou and Soumousso (localities where breeding is possible temporarily only), the former accumulating larger amounts of ascorbic and lactic acids, glucose and maltose. The accumulation of lactic acid could depict the severe osmotic and/or hypoxic stress endured by these females under ODS conditions, thus eliciting anaerobic metabolism [53]. Alternatively, the increased amounts of lactic acid could be used for glucose synthesis, whose levels were increased in ODS-reared specimens from Déou only. Altogether, these specific physiological changes are consistent with the higher level of stress experienced by these mosquitoes thriving in temporary breeding sites and subjected to severe dry season, and add supporting evidence to the existence of “strong” *versus* “weak” aestivating strategies in *An. coluzzii* [20].

Expression levels of both *AKH-I* and *ACP* (formerly called AKH-II) mRNA increased in ODS-reared females from Déou and Soumousso, respectively, i.e. in two localities where water collections quickly dry up at the onset of the dry season. There is evidence that both AKH-I and ACP peptides may have different roles [28, 54, 55], and are differently expressed inside insects. AKH-I is mainly detected from heads and thoraces in female *An. gambiae*, whereas ACP was only detected from the heads. The exact physiological functions of ACP peptides have not yet been described in anopheline species. However, Hansen et al. [56] showed that ACP peptides do not activate receptors of AKH or corazonin in anopheline species. Accordingly, these authors suggested that ACP is a structural intermediate of AKH and corazonin peptides. More generally, the roles of AKH peptides are well studied in arthropods, and two main modes of action were proposed in insects [57]. First, AKH peptides may contribute to the increase of locomotor activities [58] and inhibition of the synthesis of vitellogenin [59]. Secondly, these peptides stimulate the activity of the intermediary metabolism within the fat body, resulting in hyperlipemia, hyperglycemia, hypertrehalosemia and cardioaccelaration. Finally, recent works suggested that increased levels of AKH peptides are involved in the adjustment of the energetic and nutrient homeostasis in insects entering overwinter diapause (see [10, 60]). Thus, increased expression level of *AKH-I* in ODS-reared females from Déou could provide further support to the “strong aestivation” theory in these specimens.

## Conclusions

Recent reviews demonstrated the difficulty to detect and describe “conventional” aestivating phenotype(s) in anopheline mosquitoes [17, 61]. In the present study, we assessed the seasonal phenotypic plasticity of *An. coluzzii* populations, a main malaria vector in West Africa, using a variety of biochemical and molecular markers. We confirmed the existence of distinct physiological and biochemical rearrangements at the onset of the dry season among populations. Interestingly however, results did not show strong patterns of variations following the larval ecotype (permanent *vs* temporary breeding site) and the geographical origin (north *vs* south-western) of mosquitoes. Our physiological data do support the hypothesis that different aestivation abilities may exist among populations of *An. coluzzii* inhabiting contrasted ecological settings, as previously suggested [19, 20]. In particular, the striking metabotypes differentiation and the high *AKH* mRNA expression level observed in females from Déou may suggest the existence of a “strong” aestivation strategy in these specimens. Overall, these results highlight the complexity of the dry season survival strategies exhibited by malarial mosquitoes at local levels. Beyond pure academic interest, this work provides novel insights into the evolutionary biology and the dry season ecology of *An. coluzzii*, a major vector of human malaria. Our study highlights also that the development and implementation of innovative vector control strategies have to be explored taking into account the local phenotypic variations of mosquito populations.

## Additional files

Additional file 1: Table S1. Nucleotide sequences of the primers used in qRT-PCR reactions for the amplification of Actin, Rpl13, Rpl7, Rpl5, NADPH, hsp83, h3a, and 18s, *AKH-I, ACP* in *An. gambiae*. (DOCX 9 kb)
Additional file 2: Table S2. List of the 36 metabolites detected in females of *Anopheles coluzzii*. (DOCX 9 kb)
Additional file 3: Supplementary dataset 1. Raw data for protein, triglyceride and metabolomics assays. (XLSX 51 kb)

## Abbreviations

ACP: AKH/corazonin-related peptide
AKH: Adipokinetic hormones
F6P: Fructose 6-phosphate
G6P: Glucose 6-phosphate
LD1: First axis of the linear discriminant analysis
LD2: Second axis of the linear discriminant Analysis
LDA: Linear discriminant analysis
ODS: Onset of the dry season
PPP: Pentose phosphate pathway
RH: Relative humidity
RS: Rainy season
T0: One-hour-old females
T2: Two-day-old females
T7: Seven-day-old females
