# Supplementary material for: Comparative physiological plasticity to desiccation in distinct populations of the malarial mosquito Anopheles coluzzii
Source: Parasit Vectors. 2016 Nov 2;9:565. doi: 10.1186/s13071-016-1854-1 (PMC5094013; doi:10.1186/s13071-016-1854-1)
Supplement: Additional file 1: Table S1. — Nucleotide sequences of the primers used in qRT-PCR reactions for the amplification of Actin, Rpl13, Rpl7, Rpl5, NADPH, hsp83, h3a, and 18s, AKH-I, ACP in An. gambiae. (DOCX 9 kb) [file 13071_2016_1854_MOESM1_ESM.docx]

| **Primer** | **Direction** | **Séquences (5'-3')** |
| --- | --- | --- |
| *Actin* | FOR | CTGGACTTCGAGCAGGAGAT |
| *Actin* | REV | CGCACTTCATGATCGAGTTG |
| *Rps13* | FOR | TATTTCCAAATCCGCGCTAC |
| *Rps13* | REV | CATGATACGCAGCACCTTGT |
| *Rps7* | FOR | ACCCCAACAAGCAGAAGAGA |
| *Rps7* | REV | TACACCGACGCAAAAGTGTC |
| *Rpl5* | FOR | GGACTGAACATTCCGCACTC |
| *Rpl5* | REV | GATGCCCAGCGAGATGTACT |
| *h3a* | FOR | ATCCGTCGGTACCAGAAGTC |
| *h3a* | REV | AATGTCCTTCGGCATAATGG |
| *CytP450* | FOR | TACCAAATGAAGGGCATGGT |
| *CytP450* | REV | AACACCGCGTAATTCAAACC |
| *Tubulin* | FOR | AAGCTCGAATTCGCCATCTA |
| *Tubulin* | REV | CCAATCAAACGGTTCAGGTT |
| *hsp83* | FOR | CTGCGTGAGTTGATCTCGAA |
| *hsp83* | REV | ATCGTTCCGAGGTTGTTCAC |
| *EGFR* | FOR | GGGAATGTTGCCATCTGTTC |
| *EGFR* | REV | GACATTTCCGTACGCAGGTT |
| *18s* | FOR | ACCCGCGTCACTACAAAATC |
| *18s* | REV | CGGTAGTTTTCGTGTGCTGA |
| *AKH-I* | FOR | TGCTGATTTGTGCCTCTTTG |
| *AKH-I* | REV | ATTCCCCAACCCTACCTGAA |
| *ACP* (*AKH-II*) | FOR | CGCTGGACAGGTAACGTTTT |
| *ACP* (*AKH-II*) | REV | GACTCATCCGTTTGCAGTGA |

**Table S1.** Nucleotide sequences of primers used in qPCR reactions for the amplification of Actine, Rpl13, Rpl7, Rpl5, NADPH, hsp83, h3a, and 18s, *AKH-I, ACP* in *An. gambiae*.
