# Supplementary material for: Comparative physiological plasticity to desiccation in distinct populations of the malarial mosquito Anopheles coluzzii
Source: Parasit Vectors. 2016 Nov 2;9:565. doi: 10.1186/s13071-016-1854-1 (PMC5094013; doi:10.1186/s13071-016-1854-1)
Supplement: Additional file 2: Table S2. — List of the 36 metabolites detected in females of Anopheles coluzzii. (DOCX 9 kb) [file 13071_2016_1854_MOESM2_ESM.docx]

**Table S2.** List of the 36 metabolites detected in females of *Anopheles coluzzii*.

| Compound abbreviations in brackets |
| --- |
| **Free amino acids** |
| Alanine (Ala) |
| Glutamic acid (Glu) |
| Glycine (Gly) |
| Isoleucine (Ile) |
| Leucine (Leu) |
| Lysine (Lys) |
| Methionine (Met) |
| Phenylalanine (Phe) |
| Proline (Pro) |
| Serine (Ser) |
| Threonine (Thr) |
| Valine (Val) |
| **Sugars** |
| Fructose |
| Fructose-6-phosphate |
| Glucose |
| Glucose-6-phosphate |
| Maltose |
| Ribose |
| Trehalose |
| **Polyols** |
| Adonitol |
| Arabitol |
| Glycerol |
| Glycerol-3-phosphate |
| Inositol |
| Mannitol |
| Sorbitol |
| Xylitol |
| **Intermediate metabolites** |
| Succinic acid |
| **Other metabolites** |
| Ethanolamine |
| Ascorbic acid |
| Cadaverine |
| Gamma-aminobutyric acid (GABA) |
| Gluconic acid |
| Glyceric acid |
| Lactic acid |
| Phosphoric acid |
